# A method for visualizing surface-exposed and internal PfEMP1 adhesion antigens in *Plasmodium falciparum *infected erythrocytes

**DOI:** 10.1186/1475-2875-7-101

**Published:** 2008-06-05

**Authors:** Dominique Bengtsson, Kordai M Sowa, Ali Salanti, Anja TR Jensen, Louise Joergensen, Louise Turner, Thor G Theander, David E Arnot

**Affiliations:** 1 Centre for Medical Parasitology, Department of International Health, Immunology & Microbiology, Faculty of Health Sciences, University of Copenhagen and Department of Infectious Diseases, Copenhagen University Hospital (Rigshospitalet), CSS Øster Farimagsgade 5, Bygning 22 & 23, Postboks 2099, 1014 Copenhagen K, Denmark; 2 Institute of Infection and Immunology Research, School of Biology, University of Edinburgh, West Mains Road, Edinburgh, EH9 3JT, Scotland, UK

## Abstract

**Background:**

The insertion of parasite antigens into the host erythrocyte membrane and the structure and distribution of *Plasmodium falciparum *adhesion receptors on that membrane are poorly understood. Laser scanning confocal microscopy (LSCM) and a novel labelling and fixation method have been used to obtain high resolution immuno-fluorescent images of erythrocyte surface PfEMP1 and internal antigens which allow analysis of the accumulation of PfEMP1 on the erythrocyte membrane during asexual development.

**Methods:**

A novel staining technique has been developed which permits distinction between erythrocyte surface PfEMP1 and intracellular PfEMP1, in parasites whose nuclear material is exceptionally well resolved. Primary antibody detection by fluorescence is carried out on the live parasitized erythrocyte. The surface labelled cells are then fixed using paraformaldehyde and permeabilized with a non-ionic detergent to permit access of antibodies to internal parasite antigens. Differentiation between surface and internal antigens is achieved using antibodies labelled with different fluorochromes and confocal microscopy

**Results:**

Surface exposed PfEMP1 is first detectable by antibodies at the trophozoite stage of intracellular parasite development although the improved detection method indicates that there are differences between different laboratory isolates in the kinetics of accumulation of surface-exposed PfEMP1.

**Conclusion:**

A sensitive method for labelling surface and internal PfEMP1 with up to three different fluorochromes has been developed for laser scanning confocal optical microscopy and the analysis of the developmental expression of malaria adhesion antigens.

## Background

*Plasmodium falciparum *malaria parasites radically alter the host erythrocyte's membrane structure and adhesion properties [1,2]. Mature parasitized cells withdraw from the peripheral circulation to a variety of endothelial sequestration sites, after malaria parasite adhesion receptors are inserted into the erythrocyte membrane [2-4]. Blocking this cytoadhesion reaction and reversing sequestration is a potential malaria vaccination strategy to alleviate the pathology caused by tissue sequestration and force adhesion-blocked parasites to carry out their entire development in the hostile environment of the spleen-filtered peripheral circulation [5]. The best understood malaria adhesins are the PfEMP1 proteins. Their transport to the erythrocyte membrane has been the subject of many studies although a comprehensive model for the transport and insertion of PfEMP1 antigens into the erythrocyte membrane has not yet emerged [6-9].

Laser scanning confocal microscopic (LSCM) visualization of the distribution of fluorescently labelled antigens of malaria parasites is limited by the fact that identification of surface-exposed epitopes requires antibody binding to intact cells but detection of intracellular proteins involves fixation and permeabilization. The use of organic solvents and detergents to achieve this can lead to protein extraction and denaturation [10-12]. New epitopes also become exposed after denaturation and this increases the risk of non-specific antibody cross-reactivity, a frequently encountered problem in malaria immunology and cell biology [13].

This study reports a method for combining surface antigen labelling of live cells with subsequent internal antigen staining. The technique gives excellent preservation of internal parasite structures such as the dividing nuclei and achieves improved distinction between of surface and internal antibody staining. Antibodies against two well-characterized PfEMP1 antigens and the erythrocyte glycophorin A antigen have been used label PfEMP1 antigens on the surface of live *P. falciparum *infected erythrocytes and validate the specificity of the method.

## Methods

### *Plasmodium falciparum *lines

Indirect immuno-fluorescence assays (IFA) were performed on clonal lines of *P. falciparum*, genotyped regularly using nested GLURP and MSP2-specific primers in a single PCR reaction [14]. Continuously cultured parasites of the FCR3 lineage are referred to as FCR3 (unselected) parasites. The FCR3/BeWo parasites are so named because they have been pre-selected for adhesion to human syncytiotrophoblastic BeWo cells [15]. To obtain cells expressing the *var2csa *PfEMP1 gene and the corresponding VAR2CSA protein, 3D7*var4 *cells expressing the VAR4 PfEMP1 antigen were selected using streptavidin-coated magnetic Dynabeads^® ^and biotin-labelled antibodies, to capture parasites reacting with surface-binding antibodies [16]. Antibodies used to select 3D7*var4 *cells were raised in rabbits immunized with a recombinant fragment of the VAR4-CIDRα PfEMP1 (PFD1235w).

### Antibodies

A monoclonal antibody against glycophorin A (mouse anti-human CD235a) was purchased from BD Biosciences. Rabbit antisera against baculovirus-produced recombinant domains of the VAR2CSA PfEMP1 protein (VAR2 antisera) have been described [17,18]. VAR4 antisera are derived by immunizing rabbits with a recombinant antigen containing both the DBL4 and DBL5 domains of the *var4 *gene [16,19]. Antisera were depleted of antibodies reacting with human red cell antigens by mixing equal amounts of rabbit antiserum with human O^+ ^erythrocyte suspension in 2% FCS in PBS, then incubating the mixture at 4°C for one hour. Affinity purified rabbit antiserum against the conserved sequence DITSSESEYEELDINDIC, present in the intracellular (Exon 2) domain of all known PfEMP1 proteins, was used in the co-staining experiments [20].

### Live parasite surface staining

Magnetic cell-sorted mature parasites were washed three times in 1% BSA (Sigma) in PBS. For surface antigen staining, two microlitres of the parasite pellet was mixed with 100 microlitres of a 1/250 dilution of anti-PfEMP1 antibody and/or a 1/10,000 dilution of anti-glycophorin A antibody. The primary antibody incubation was for 30 mins at 4°C. The secondary incubation with Alexa-conjugated anti-mouse or anti-rabbit IgG (diluted 1/2000) was for 30 mins at 4°C, after which surface stained cells are ready for the internal staining procedures.

### Fixation and permeabilization of the surface labelled cells

Ten microlitres (10%) of the surface labelled cell suspension was air dried onto a multi-well microscope slide (Diagnostika). Cells were fixed by covering the surface with a 4% paraformaldehyde solution for 10 mins. The formaldehyde fixative was washed off with PBS and the slide incubated in a 0.1% Triton X-100 permeabilization solution for five minutes. The permeabilization solution was again washed off with PBS prior to incubating the slide in a blocking solution of 1% PBS/BSA for 30 mins. All steps were carried at 4°C.

### Post-fixation staining

Fixed, permeabilized cells were incubated with all antibodies targeted to internal antigens (in these experiments, internal PfEMP1) for one hour at 4°C in a humid box kept in the dark. For routine experiments Alexa^®^564 labelled anti-IgG (1/2000) and DAPI (10 ng/ml) were used. In the validation experiments requiring specific surface staining with two fluorochromes and internal staining of a third antigen, Pacific Blue staining (1/500) was used as the internally staining fluorochrome. IFA slides mounted with anti-fade reagent (Invitrogen) were visualized on a confocal microscope.

### Confocal microscopy

All images were taken using a model TE 2000-E Nikon Eclipse confocal microscope, with 60 × (numerical aperture 1.4) Apoplan oil immersion objective lens, equipped with a Differential Interference Contrast (DIC) beam shearing interference contrast-enhancing system. The quality of the slide, the parasite preparation, labelling and background were checked using a fluorescence microscope with fluorescence-free immersion oil (Fluka). Images were captured using the EZ-C1 Gold imaging system (version 3.30). Images of the labelled cells were captured using the Frame Lambda channels, with gain adjusted manually for each laser (450/35, 515/30 and 605/75). Pixel Dwell was 4–6 m^-1^s^-1^, with steps 512 × 512 pixels. The images were averaged on seven scanned images using the normal scan mode.

Merged images were captured using the Trans and Frame Lambda channel devices. Gain was adjusted manually for each laser. Optical sectioning for the *z *direction imaging was carried out in either 150 nanometre steps. Images were saved in IDS software format and processed using Adobe Photoshop software for sharpening edges, contrast and light adjustment. Images are displayed with the 5 μm scale bar calculated by the EZ-C1 software, except the fluorescence alone or 'plane of best focus' fluorescence images of infected erythrocytes, where the scale is not calculated.

## Results

### Optimized fixation and staining for immuno-fluorescence microscopy of *P. falciparum *infected erythrocytes

Light microscopy with malaria parasites has traditionally used Giemsa's modified Romanowsky stain, fixing the PE to the glass slide with methanol. This long established methodology may not be optimal for confocal immuno-fluorescence microscopy and reported contra-indications on the use of methanol and acetone fixation [10,12] led us to compare methanol or methanol/acetone fixation with paraformaldehyde fixation/Triton X-100 permeabilization protocols.

No pronounced difference in capacity to immuno-detect surface or internally labelled antigen was noted in comparative fixations, although particulate background fluorescence was a persistent problem following acetone fixation. Using non-ionic detergent for cell permeabilization improved fluorescence signals and reduced non-specific background fluorescence. The paraformaldehyde/Triton X-100 protocol described above was therefore adopted. In avoiding methanol and acetone in favour of paraformaldehyde fixation, this protocol is similar to another recently published method for improving the preparation of *P. falciparum *infected erythrocytes for microscopy [21], but uses low concentrations of non-ionic detergent rather than glutaraldehyde to enhance antibody binding and reduce background staining.

### Validation of antibody and labelling specificity when staining live *P. falciparum*-infected erythrocytes selected for the expression of particular PfEMP1 antigens

**Figure 1:**
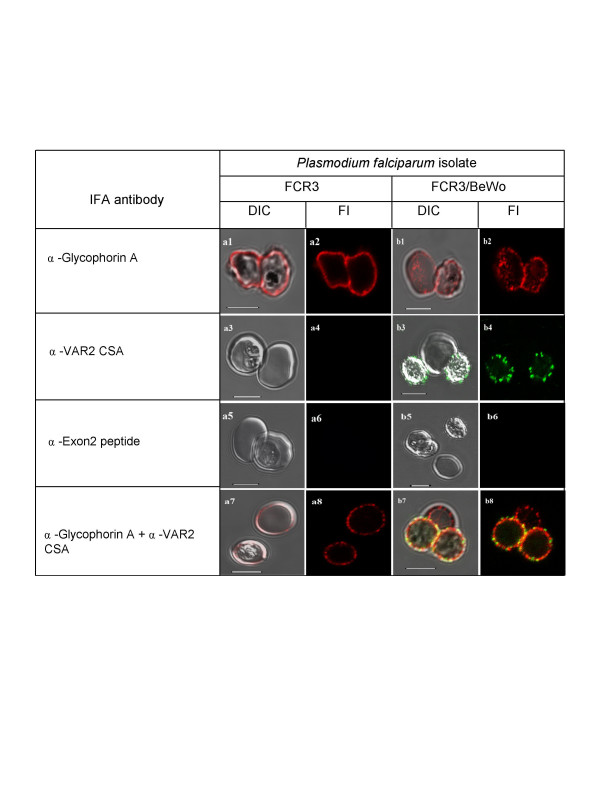
**Specific fluorescent antibody labelling of surface antigens on live *P. falciparum*-infected erythrocytes selected for cytoadhesion**. All plates in the odd numbered plates show the DIC shadow-cast image of with the fluorescence image superimposed. All even numbered plates show the fluorescence image alone. The *a *series shows antibody binding to unselected *P. falciparum *FCR3 infected erythrocytes. Plates in the *b *series show *P. falciparum *FCR3 infected erythrocytes which have been pre-selected for adhesion to BeWo cells. Before preparation for microscopy magnetically purified cells were seeded with control uninfected erythrocytes. The antibodies used in each row of images are indicated on the left. Scale bar 5 μM.

A monoclonal antibody against glycophorin A, was used as a control antibody for surface staining of a host erythrocyte membrane protein. There are around a million copies of the glycophorin A 36 kDa trans-membrane sialoglycoprotein on the red blood cell surface [22]. Figure 1 (plates a1 and a2) shows live staining of two intact infected erythrocytes of unselected FCR3 with the anti-Glycophorin A antibody, detected with Alexa^®^568-conjugated anti-mouse IgG. Glycophorin A staining appears uniform across the surface of these intact, infected erythrocytes. Figure 1 (Plates a3 and a4) shows the negative reaction of infected and uninfected erythrocytes with an antiserum specific for the VAR2CSA protein. Figure 1 (Plates a5 and a6) shows that intact, infected erythrocytes cannot be stained with an antibody recognizing the Exon 2-encoded intracellular domain of PfEMP1 proteins. Figure 1 (Plates a7 and a8) shows that incubating unselected FCR3 with both anti-glycophorin A and anti-VAR2CSA antibodies results in only glycophorin A fluorescence. The reaction is negative with the anti-VAR2CSA antiserum because this PfEMP1 antigen is not expressed by the great majority (99.5%) of unselected FCR3 cells [23].

Specific detection of the VAR2CSA PfEMP1 on FCR3/BeWo cells is also shown in Figure 1. Both infected (plate b1, right cell), and uninfected erythrocytes (plate b1, left cell) are intact and recognized by the anti-glycophorin A antibody. Plates b3 and b4 illustrate the specific reactivity of the anti-VAR2CSA antibody with the surface of FCR3/BeWo cells. Plates b5 & b6 show that the intra-cellular PfEMP1 domain is, as expected, not accessible to the anti-Exon 2 antiserum in live, infected erythrocytes (the bottom right cell in plate b5 is uninfected). Plates b7 & b8 show that co-incubating FCR3/BeWo with anti-glycophorin A and anti-VAR2CSA antibodies results in double staining of the infected erythrocytes but glycophorin A staining alone on the uninfected erythrocyte (centre top in plates b7 & b8). Due to the proximity of host and parasite surface antigens on the erythrocyte membrane, there is significant co-localization of the two fluorochromes (rendered as yellow 'false' staining), the result of both Alexa^®^568 (Glycophorin A) and Alexa^®^488 (VAR2CSA) derived photons being detected in the same image pixels.

### Validation of antibody and labelling specificity of co-stained surface and internal PfEMP1

**Figure 2:**
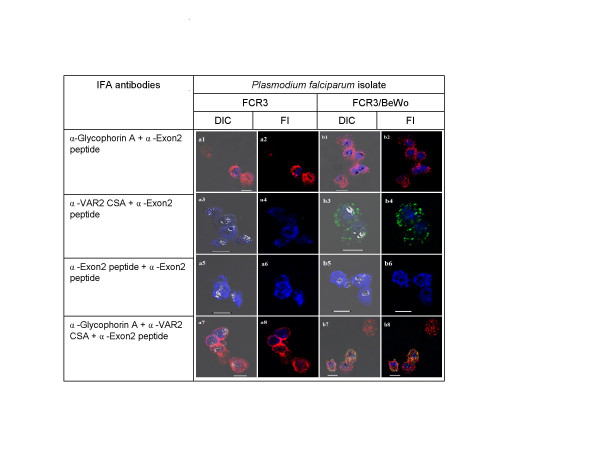
**Specific co-staining of *P. falciparum *infected erythrocyte surface and internal antigens**. All plates in the odd numbered plates show the DIC shadow-cast image of with the fluorescence image superimposed. All even numbered plates show the fluorescence image alone. The *a *series shows antibody binding to unselected *P. falciparum *FCR3 infected erythrocytes. Plates in the *b *series show *P. falciparum *FCR3 infected erythrocytes which have been pre-selected for adhesion to BeWo cells. Before preparation for microscopy magnetically purified cells were seeded with control uninfected erythrocytes. The antibodies used in each row of images are indicated on the left. Scale bar 5 μM.

Figure 2 illustrates experiments validating the specific surface and internal anti-PfEMP1 antibodies and staining procedure. Two erythrocyte surface staining antibodies were used, anti-glycophorin A (red) and anti-VAR2CSA (green). Internal antigens, detectable only after fixation and permeabilization of the surface labelled cells were visualized using an anti-PfEMP1 Exon 2 antibody (Pacific Blue stain).

Figure 2, plates a1 & a2, shows the 'DIC plus fluorescence' and 'fluorescence alone' images of one uninfected and two infected erythrocytes (*P. falciparum *FCR3/unselected). All erythrocyte surfaces are glycophorin A-positive and all infected erythrocytes show internal anti-Exon 2 staining. Because this antibody binds a conserved epitope in the intra-cellular domain of all PfEMP1 antigens, it detects any PfEMP1 antigen synthesized by a *P. falciparum*-infected cell. Glycophorin A/Exon 2 co-staining of FCR3/BeWo cells expressing surface VAR2CSA protein (plates b1 & b2) thus shows the same internal staining phenotype as that seen in unselected but parasitized erythrocytes.

When surface staining for the VAR2CSA antigen, unselected FCR3 infected erythrocytes are negative, although they are positive for internal PfEMP1 antigen (Figure 2) (plates a3 and a4). By contrast, FCR3/BeWo parasites are surface stained by the anti-VAR2CSA antibody (plates b3 & b4). Absence of surface reactivity of the anti-Exon 2 antibody with all parasitized erythrocytes is confirmed in plates a5-a6 & b5-b6.

**Figure 3:**
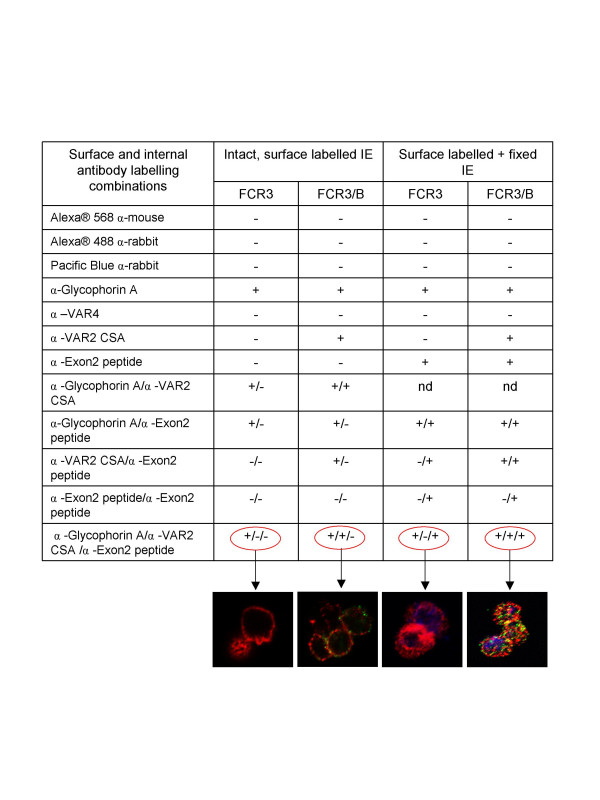
Summary of the validation experiments demonstrating specific labelling and distribution of surface and internal PfEMP1 antigens in *Plasmodium falciparum *infected erythrocytes.

Triple staining of surface and internal antigens can also be achieved. Figure 2 (plates a7-a8) shows unselected FCR3 infected erythrocytes reacted with the two surface-specific antibodies followed by internal staining of intracellular PfEMP1. These cells are positive for glycophorin A, negative for VAR2CSA and positive for internal PfEMP1 antigens. By contrast, FCR3/BeWo parasites are positive for VAR2CSA (green), glycophorin A (red) and Pacific Blue (internal PfEMP1). Since glycophorin A and VAR2CSA co-localize to the FCR3/Bewo infected erythrocyte surface membrane, yellow false colouring indicates emission from both fluorochromes being detected in the same image pixels. The results of all the methodological validation experiments are summarized in Figure 3.

### Confocal optical sectioning infected erythrocytes with fluorescence labelled VAR2CSA and ethidium bromide stained DNA

**Figure 4:**
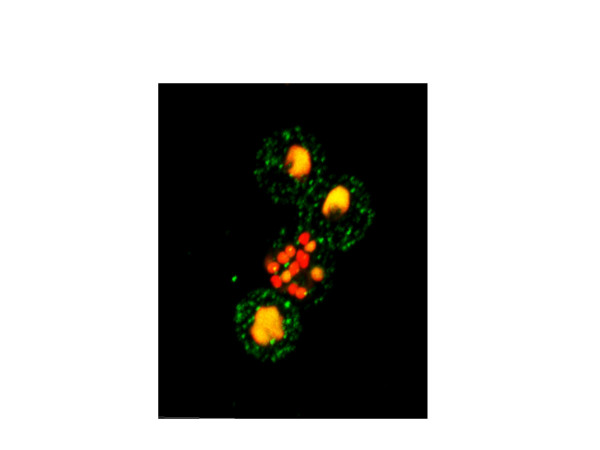
**Optical sectioning after surface PfEMP1 labelling and nuclear DNA staining**. The 'plane of best focus' of four FCR3 BeWo adhesion selected *P. falciparum *infected erythrocytes, surface labelled with anti-VAR2CSA and nuclear-stained with ethidium bromide. This image constitutes the composite image of the series of optical section images shown in Figure 5.

**Figure 5:**
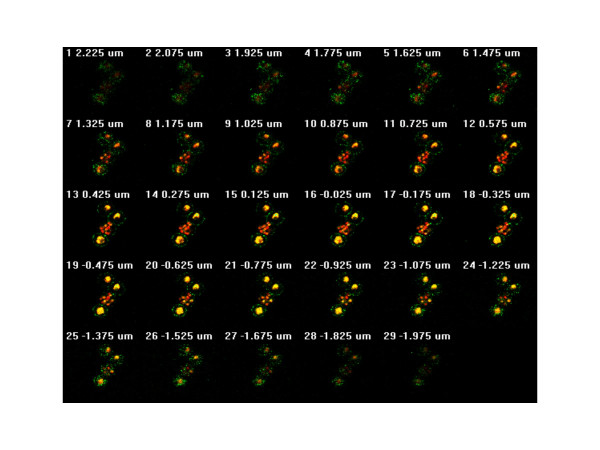
**Successive confocal optical sections focussing through surface labelled, intact *P. falciparum *cells**. Serial confocal optical sections, taken in 150 nanometer steps, moving through the live *P. falciparum *(FCR3/BeWo) infected erythrocyte, surface-labelled with fluorescent secondary antibodies recognising the anti-VAR2CSA antibodies, in the presence of the membrane permeable nuclear dye ethidium bromide.

Figure 4 illustrates the resolution of surface detectable PfEMP1 on the erythrocyte membrane obtained using this methodology to assess membrane loading of PfEMP1 during asexual parasite development. The image shows the 'surface of best focus' [24] of four *P. falciparum *infected (FCR3/BeWo) erythrocytes surface stained with anti-VAR2CSA antibodies in the presence of ethidium bromide, allowed to settle onto a poly-L-lysine coated microscope slide. This picture is a composite of 150 nanometre spaced optical sections carried out using the *z*-dimensional stacking function of the microscope. Each optical section, from the basal erythrocyte surface adhering to the poly-L-lysine coated slide, up through the cell to the top surface, is shown in Figure 5.

The photonic emission from laser-excited fluorochrome on the detection antibody, varies in intensity as the inverse of the square of the distance from the source to the detector. The intensity of light emitted from point sources on the erythrocyte surface membrane thus increases as the distance to the optical plane decreases and diminishes as the point source recedes during the optical sectioning. There is no internal labelling of the intact parasitized erythrocytes with the antibody, although the erythrocyte membrane is permeable to the nucleophilic dye, ethidium bromide. As the optical sectioning (Fig. 5) reaches the middle of the erythrocyte, the punctuate anti-PfEMP1 staining marks out a circumference, giving the characteristic 'necklace-of-beads' pattern of staining associated with fluorescence labelled anti-PfEMP1 antibodies reacting with punctuate, dispersed structures on erythrocyte surface membrane.

Division of the parasite nuclear material is exceptionally well displayed in Figure 4. This helps in correlating the relationship between the development of the parasite and the amount of surface expression of PfEMP1 (see also Fig. 5). The top two infected erythrocytes shown in Figure 4 are relatively early stage trophozoites. The bottom cell is a late trophozoite/early schizont in which four distinct nuclei are about to become separate. The lower middle cell is clearly a late schizont, in which 13–14 nuclear bodies are in the process of what appears to be near-synchronous division. Interestingly, the amount of surface labelled VAR2CSA does not appear to significantly differ between these different developmental stages of blood stage *P. falciparum*. The optical sections shown in Figure 5 demonstrate that this equivalence is not simply an artefact of the strong schizont nuclear fluorescence masking surface PfEMP1-derived fluorescence, as the equivalence of staining intensity is a surface property which is visible before interference by nuclear fluorescence becomes apparent.

### Surface PfEMP1 accumulation during parasite development

**Figure 6:**
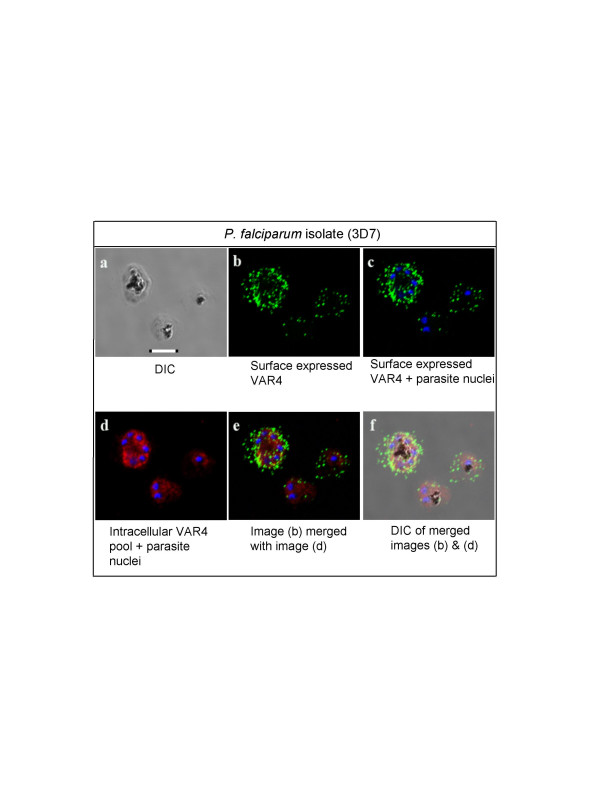
**Visualizing surface Var 4 PfEMP1 at different stages of parasite development**. Surface and internal staining of Var4-type PfEMP1 antigens on the erythrocyte surface and within the parasitized cell. 3D7 infected erythrocytes are shown at three different stages of development with different amounts of surface labelled Var4 PfEMP1 (green Alexa^®^488 fluorescence). Red Alexa^®^568 fluorescence indicates intracellular reaction with the anti-PfEMP1 antisera. Nuclei are stained with DAPI. Scale bar 5 μM.

Figure 6 (plate 6a) shows the DIC images of three parasitized erythrocytes (3D7 lineage) containing, from right to left, progressively larger haemozoin granules. These cells have been surface stained as live cells, then fixed for visualization of surface and internal and internal antigen staining. Plate 6c shows the blue DAPI-stained parasitized cells are, from left to right, uninucleate, binucleate and hexanucleate. The two methods of estimating the developmental stage correlate well and the fixed *P. falciparum*-infected erythrocytes are clearly an early trophozoite, an early schizont and a mid-stage schizont, respectively.

Plate 6b shows the results of live staining the cells before fixation with anti-VAR4 antibodies to a Group A type-PfEMP1, followed by internal staining with the same polyclonal anti-PfEMP1 antiserum. Green fluorescence indicates Alexa^®^488 labelled, surface-exposed Group A-type PfEMP1. The resolution of the surface fluorescent staining allows comparison of the stage of parasite development with the amount of surface exposed PfEMP1. Plate 6c shows the PfEMP1 surface stained image merged with the DAPI nuclear stained fluorescence image. These images, like those shown in Figure 4, indicate that the amount of surface detectable PfEMP1 is often not directly related to morphological markers of the age of the parasite. Uninucleate infected cells can have as much or more surface PfEMP1 than dinucleate early schizonts (Figure 6b). However, in these infected erythrocytes (expressing *Var *4-type PfEMP1), unlike those shown in Figure 4 (FCR3/BeWo expressing VAR2CSA) there is clearly more surface PfEMP1 on the membrane of the schizont, compared to the trophozoites.

Figure 6 also shows fluorescence derived from antibodies reacting with internal sources of PfEMP1 in the fixed cells. Plate 6d shows the results of staining with polyclonal anti-VAR4 antibody, with intracellular reactivity detected using Alexa^®^568 red conjugated secondary antibody. The secondary staining of intracellular PfEMP1 indicates that most of this material may still be within the parasitophorous vacuole membrane, even in schizonts, although more marker antigens are required to confirm this localization. Plates 6e and 6f show merging of the Alexa^®^488 green surface PfEMP1 staining with the Alexa^®^568 red intra-cellular PfEMP1 staining, superimposed on the fluorescence and DIC images respectively.

## Discussion

This novel fluorescence labelling method gives improved detection and resolution of erythrocyte surface exposed antigens and a tool to correlate surface antigen distribution with intracellular antigen distribution. The use of antibodies against VAR2CSA and a Var4 type PfEMP1 confirms that surface-exposed epitopes of these PfEMP1 antigens first appear on the surface of the infected erythrocyte membrane in the early trophozoite stages of both the FCR3 and 3D7 parasites used in this study. However, it remains unclear for how long PfEMP1 continues to be deposited into the erythrocyte membrane and how the cytoadhesion complex is actually assembled.

It has been proposed that the surface distribution of PfEMP1 on the erythrocyte membrane is not a continuous process but limited to a period following the onset of trophozoite stage. Such a 'trophozoite-limited' PfEMP1 loading model was first proposed to explain data showing a lack of replenishment of surface PfEMP1 following surface trypsinization [25] In the present study, FCR3/BeWo parasites did not show a significant increase in the amount of the VAR2CSA PfEMP1 present on the erythrocyte surface, between early trophozoite and late schizont stages (Figure 4). This observation is consistent with the hypothesis of a 'trophozoite-limited' and relatively narrow time window of PfEMP1 membrane loading.

However, the results of the study on surface exposure of Var4 type PfEMP1 on 3D7 lineage cells show a different relationship between parasite stage and amount of surface PfEMP1. In these experiments, an approximate equality in the amount of surface-exposed PfEMP1 present on all post-trophozoitic stages was not observed (Figure 6). Older schizonts showed considerably more surface PfEMP1. This implies that, at least for *var4 *PfEMP1 loading on 3D7 lineage parasites, the process is more continuous and continues into the later stages of development of *P. falciparum*. This opens the possibility that there are isolate and/or PfEMP1 antigen-specific differences in the kinetics of surface adhesin appearance.

These experiments with this novel surface antigen labelling methodology indicate that a degree of uncertainty exists in our knowledge of parasite adhesion, which has implications for malaria pathogenesis and adhesion-blocking therapies. During the course of malaria parasite development, is there a short phase of deposition of PfEMP1 antigen onto the erythrocyte surface which rapidly precipitates tissue sequestration? Or alternatively, is there a longer period of membrane loading of PfEMP1, continuing after endothelial sequestration? The former implies that adhesion antigens may not be a long-lived target for adhesion blocking antibodies. The latter would imply that increasing quantities of surface PfEMP1 and increasing numbers of adhesion complexes may be necessary to maintain sequestration, as the mass of the developing parasite increases during schizogony. It may be necessary to move beyond the idiosyncratic behaviour of long term laboratory cultured isolates and investigate these issues in *P. falciparum *samples taken directly from human clinical infections.

## Conclusion

A novel method for combining surface labelling of live, intact cells with subsequent fixation and permeabilization for internal staining achieves significantly improved resolution of fluorescence-labelled surface and intracellular antigens in *P. falciparum *infected erythrocytes.

## Authors' contributions

DEA and DB conceived the study, which was largely carried out by DB. KMS helped design the studies and carried out parasite culture and manipulation. AS, ARTJ, LJ, LT and TGT discussed experiments and results and provided antibodies and reagents. DEA wrote and then edited the manuscript. All authors discussed the data and helped finalize the manuscript. All authors read and approved the final manuscript.

## References

[B1] Cooke BM, Lingelbach K, Bannister LH, Tilley L (2004). Protein trafficking in *Plasmodium falciparum*-infected red blood cells. Trends Parasitol.

[B2] Wickham ME, Rug M, Ralph SA, Klonis N, McFadden GI, Tilley L, Cowman AF (2001). Trafficking and assembly of the cytoadherence complex in *Plasmodium falciparum*-infected human erythrocytes. Eur Mol Biol Organ J.

[B3] Knuepfer E, Rug M, Klonis N, Tilley L, Cowman AF (2005). Trafficking of the major virulence factor to the surface of transfected *P. falciparum*-infected erythrocytes. Blood.

[B4] Maier AG, Rug M, O'Neill MT, Beeson JG, Marti M, Reeder J, Cowman AF (2007). Skeleton-binding protein 1 functions at the parasitophorous vacuole membrane to traffic PfEMP-1 to the *Plasmodium falciparum*-infected erythrocyte surface. Blood.

[B5] Hviid L, Salanti A (2007). VAR2CSA and protective immunity against pregnancy-associated *Plasmodium falciparum *malaria. Parasitology.

[B6] Lingelbach K, Przyborski JM (2006). The long and winding road: protein trafficking mechanisms in the *Plasmodium falciparum *infected erythrocyte. Mol Biochem Parasitol.

[B7] Papakrivos J, Newbold CI, Lingelbach K (2005). A potential novel mechanism for the insertion of a membrane protein revealed by a biochemical analysis of the *Plasmodium falciparum *cytoadherence molecule PfEMP-1. Mol Microbiol.

[B8] Tilley L, McFadden G, Cowman A, Klonis N (2007). Illuminating *Plasmodium falciparum*-infected red blood cells. Trends Parasitol.

[B9] Cooke BM, Buckingham DW, Glenister FK, Fernandez KM, Bannister LH, Marti M, Mohandas N, Coppel RL (2006). A Maurer's cleft-associated protein is essential for expression of the major malaria virulence antigen on the surface of infected red blood cells. J Cell Biol.

[B10] Hoetelmans RWM, Prins FA, Cornelse-ten Velde I, Meer J van der, Velde CJH van de, van Dierendonck JH (2001). Effects of acetone, methanol or paraformaldehyde on cellular structure, visualized by reflection contrast microscopy and transmission and scanning electron microscopy. App Immunohistochem Mol Morph.

[B11] Weibull C, Christiansson A, Carlemalm E (1983). Extraction of membrane lipids during fixation, dehydration and embedding of *Acholeplasma laidlawi *cells for electron microscopy. J Microscopy.

[B12] Melan MA, Sluder G (1992). Redistribution and differential extraction of soluble proteins in permeabilized cultured cells. Implications for immunofluoresence microscopy. J Cell Sci.

[B13] Creasey AM, Staalsoe T, Raza A, Arnot DE, Rowe A (2003). Non-specific IgM and Chondroitin Sulphate A binding are linked phenotypes of *Plasmodium falciparum *isolates implicated in malaria during pregnancy. Infect Immun.

[B14] Dahlback M, Lavstsen T, Salanti A, Hviid L, Arnot DE, Theander TG, Nielsen MA (2007). Changes in *var *gene mRNA levels during erythrocyte development in two phenotypically distinct *Plasmodium falciparum *parasites. Malar J.

[B15] Haase RN, Megnekou R, Lundquist M, Ofori MF, Hviid L, Staalsoe T (2006). *Plasmodium falciparum *parasites expressing pregnancy-specific variant surface antigens adhere strongly to the choriocarcinoma cell line BeWo. Infect Immun.

[B16] Jensen ATR, Magistrado P, Sharp S, Jørgensen L, Lavstsen T, Chiucciuini A, Salanti A, Vestergaard LS, Lusingu JP, Hermsen R, Sauerwein R, Christensen J, Nielsen MA, Hviid L, Sutherland C, Staalsoe T, Theander TG (2004). *Plasmodium falciparum *associated with severe childhood malaria preferentially expresses PfEMP1 encoded by group A *var *genes. J Exp Med.

[B17] Barfod L, Bernasconi NL, Dahlback M, Jarrossay D, Andersen PH, Salanti A, Ofori MF, Turner L, Resende M, Nielsen MA, Theander TG, Sallusto F, Lanzavecchia A, Hviid L (2007). Human pregnancy-associated malaria-specific B cells target polymorphic, conformational epitopes in VAR2CSA. Mol Microbiol.

[B18] Barfod L, Nielsen MA, Turner L, Dahlback M, Jensen ATR, Hviid L, Theander TG, Salanti A (2006). *Baculovirus*-expressed constructs induce immunoglobulin G that recognizes VAR2CSA on *Plasmodium falciparum*-infected erythrocytes. Infect Immun.

[B19] Magistrado PA, Lusingu J, Vestergaard AS, Lemnge M, Lavstsen T, Turner T, Hviid L, Jensen AT, Theander TG (2007). Immunoglobulin G antibody reactivity to a Group A *Plasmodium falciparum *erythrocyte membrane protein 1 and protection from *P. falciparum *malaria. Infect Immun.

[B20] Sharling L, Sowa KMP, Thompson J, Kyriacou HM, Arnot DE (2007). Rapid and specific biotin labelling of the erythrocyte surface antigens of both cultured and *ex-vivo Plasmodium *parasites. Malar J.

[B21] Tonkin CJ, van Dooren CG, Spurck TP, Stuck NS, Good RT, Handman E, Cowman AF, McFadden GI (2004). Localization of organellar proteins in *Plasmodium falciparum *using a novel set of transfection vectors and a new immunofluorescence fixation method. Mol Biochem Parasitol.

[B22] Hassoun H, Hanada T, Lutchman M, Sahr K, Palek J, Hanspal M, Chishti AH (1998). Complete deficiency of Glycophorin A in red blood cells from mice with targeted inactivation of the Band 3 (AE1) gene. Blood.

[B23] Salanti A, Staalsoe T, Lavstsen T, Jensen ATR, Sowa MPK, Arnot DE, Hviid L, Theander TG (2003). Selective upregulation of a single distinctly structured *var *gene in chondroitin sulphate A-adhering *Plasmodium falciparum *involved in pregnancy-associated malaria. Mol Microbiol.

[B24] Pawley JB (2002). Limitations on optical sectioning in live-cell confocal microscopy. Scanning.

[B25] Kreik N, Tilley L, Horrocks P, Pinches R, Elford BC, Ferguson DJP, Lingelbach K, Newbold CI (2003). Characterization of the pathway for transport of the cytoadherence-mediating protein, PfEMP1, to the host cell surface in malaria parasite-infected erythrocytes. Mol Microbiol.

